# Supplementary material for: Validation of the Portuguese Version of the Short-Form Glasgow Composite Measure Pain Scale (CMPS-SF) According to COSMIN and GRADE Guidelines
Source: Animals (Basel). 2024 Mar 8;14(6):831. doi: 10.3390/ani14060831 (PMC10967594; doi:10.3390/ani14060831)
Supplement: Supplementary file 1 [file animals-14-00831-s001.zip › Supplementary files/Supplementary material 1 - GRADE.pdf]

Supplementary 1. Psychometric score of GRADE adapted [14, 15] to test the properties of the CMPS-SF [10]

Questions	Subtitle score	CMPS-SF [10]	CMPS-SF - Current study
**Scale development: item selection and content validation**			
**1.1. Was the process of item selection described?**	2: Scale was developed for a specific population, using a theoretical or conceptual framework, or a qualitative approach was used (e.g., consultation with clinicians or patients)<br>1: Scale was developed based on the literature review only<br>0: No information is provided about item selection	2	2
**1.2. Was content evaluated by experts? (content validation)**	2: Content was evaluated by experts in the field, a Delphi technique may have been used, and Content Validity Index (CVI) were calculated for each item included in the scale<br>1: Content was evaluated by experts, but no CVI is reported<br>0: No information is provided about content validation	1	1
**1.3. Are limitations of some items presented or discussed?**	1: No limitations or if any limitations, they are presented and item modifications have been made or precautions have been stated<br>0: No information is provided	1	1
**Subtotal-Scale Development (0 – 5)**		4/5	4/5
**Subtotal weighted score—Scale development (0–2)**		**1.6**	**1.6**
**Scale testing-reliability**			
**2.1. Was internal consistency of the scale calculated?**	2: $0.70 < \alpha < 0.90$<br>1: $0.60 < \alpha < 0.70$ or $\alpha > 0.90$ 0: $\alpha < 0.60$ or no information provided	0	2 ($\alpha = 0.7$)
**2.2. Was interrater reliability calculated?**	2: kappa > 0.60 or ICC > 0.80<br>1: 0.60 < kappa > 0.40 or 0.60 < ICC < 0.80<br>0: kappa < 0.40, ICC < 0.60 or no information provided	1	2 (ICC ≥ 0.8)
**2.3. Was interrater reliability tested with other raters besides research team?**	1: Other raters then research staff members were involved	1	1

	0: Only research staff members were involved		
**2.4. Was intrarater reliability tested? Optional—to be examined if ICC < 0.80 for interrater reliability**	2: kappa > 0.60 or ICC > 0.80<br>1: 0.60 < kappa > 0.40 or 0.60 < ICC < 0.80<br>0: kappa < 0.40, ICC < 0.60 or no information provided	0	2 (ICC ≥ 0.8)
**Subtotal—Scale development (0–5 or 0–7 if intrarater reliability testing required)**		**2/7**	**7/7**
**Subtotal weighted score—Scale development (0–6)**		1.68	6
	**Scale testing: Construct validity**		
**3.1. What is the total of participants for the purpose of testing the scale?**	2 – N > 50<br>1 – 20 > N < 50<br>0 – N < 20	0	0
**3.2. Criterion validation: Was the scale correlated with the "gold standard: measure renowned in the field of interest (e.g., the patient's self-report of pain)?**	2: r > 0.60 with the "gold standard" measure<br>1: 0.40 < r < 0.60<br>0: r < 0.40 or no information provided	0	2<br>(Unidimensional scales were considered "gold standard")
**3.3. Criterion validation: Was the sensitivity of the scale calculated?**	2: Sensitivity 80% 1: 60% Sensitivity < 80%<br>0: Sensitivity < 60% or no information provided	2	1
**3.4. Criterion validation: Was the specificity of the scale calculated?**	2: Specificity ≥80%<br>1: 60% ≤ Specificity < 80%<br>0: Specificity < 60% or no information provided	2	2
**3.5. Discriminant validation: Was the scale able to discriminate between different situations (e.g., between pain and no pain, e.g., at rest and during a nociceptive procedure, before and after the administration of an analgesic)?**	2: A clinically important difference was found<br>1: A difference was found but was not considered clinically important<br>0: No difference was found or no information is provided	2	2
**Subtotal—Scale development (0–10)**		6/10	7/10
**Subtotal weighted score—Scale development (0–8)**		4.8	5.6
**4.1. Was the feasibility (i.e., ease of usage with which clinicians can apply the instrument in the clinical setting) of the scale examined?**	1: Scale is considered to be feasible to use by more than 80% of the clinicians<br>0: Scale is considered to be complex to use by more than 20% of the clinicians or no information is provided	1	0

**4.2. Are directives of use of the scale clearly described?**	1: Yes, directives of use including the scoring method are described 0: No information about directives of use is provided	1	0
**Subtotal—Scale development (0–2)**		2/2	0/2
**Subtotal weighted score—Scale development (0–2)**		2	0
**5.1. Was the relevance of the scale or impact of its implementation in patient outcomes examined?**	1: Scale is considered to be useful and relevant to practice by more than 80% of the clinicians; use of the scale yielded a significant change into practice (e.g., better use of medication, increase in patients' assessments)<br>0: Scale is not considered to be useful and relevant to practice by more than 20% of the clinicians; use of the scale did not yield a significant change into practice or no information provided	1	0
**Subtotal—Scale development (0–1)**		0/1	0/1
**Subtotal weighted score—Scale development (0–2)**		0	0
**Total score (0–25)**		14	18
**Weighted score (0–20)**		**11.2**	**14.4**
**Quality of evidence**		Moderate	Good

Interpretation: Weighting scores 15 a 20 – Very good; 12 – 14,9 – good; 10-11.9 – Moderated; 0 – 9.9 – Few/unacceptable properties. Scales with weighting ≥ 12 showed greater validity and reliability
