# Supplementary material for: Validation of the Portuguese Version of the Short-Form Glasgow Composite Measure Pain Scale (CMPS-SF) According to COSMIN and GRADE Guidelines
Source: Animals (Basel). 2024 Mar 8;14(6):831. doi: 10.3390/ani14060831 (PMC10967594; doi:10.3390/ani14060831)
Supplement: Supplementary file 1 [file animals-14-00831-s001.zip › Supplementary files/Supplementary material 2 - ARRIVE.pdf]

The ARRIVE guidelines 2.0: author checklist

Supplementary 2. ARRIVE

The ARRIVE Essential 10

These items are the basic minimum to include in a manuscript. Without this information, readers and reviewers cannot assess the reliability of the findings.

Item		Recommendation	Section/line number, or reasonfor not reporting
**Study design**	1	For each experiment, provide brief details of study design including:<br>a. The groups being compared, including control groups. If no control group hasbeen used, the rationale should be stated.<br>b. The experimental unit (e.g. a single animal, litter, or cage of animals).	Section 2.1<br>Lines 90-92<br>Section 2.2<br>Lines 93-113
**Sample size**	2	a. Specify the exact number of experimental units allocated to each group, and thetotal number in each experiment. Also indicate the total number of animals used.<br>b. Explain how the sample size was decided. Provide details of any *a priori* samplesize calculation, if done.	Section 2.6<br>Line 161<br>Supplementary material 5<br><br>Section 2.11.<br>Lines – 202-209
**Inclusion and exclusion criteria**	3	a. Describe any criteria used for including and excluding animals (or experimental units) during the experiment, and data points during the analysis. Specify if thesecriteria were established *a priori*. If no criteria were set, state this explicitly.<br>b. For each experimental group, report any animals, experimental units or data points not included in the analysis and explain why. If there were no exclusions, state so.<br>c. For each analysis, report the exact value of *n* in each experimental group.	Section 2.2<br><br>Lines 98-113
**Randomization**	4	a. State whether randomization was used to allocate experimental units to controland treatment groups. If done, provide the method used to generate the randomization sequence.<br>b. Describe the strategy used to minimize potential confounders such as the orderof treatments and measurements, or animal/cage location. If confounders were not controlled, state this explicitly.	Section 2.8<br>Line 179-182
**Blinding**	5	Describe who was aware of the group allocation at the different stages of the experiment (during the allocation, the conduct of the experiment, the outcomeassessment, and the data analysis).	Section 2.7 – 2.11
**Outcome measures**	6	a. Clearly define all outcome measures assessed (e.g. cell death, molecular markers, or behavioural changes).<br>b. For hypothesis-testing studies, specify the primary outcome measure, i.e. theoutcome measure that was used to determine the sample size.	Section 2.11
**Statistical methods**	7	a. Provide details of the statistical methods used for each analysis, includingsoftware used.<br>b. Describe any methods used to assess whether the data met the assumptions ofthe statistical approach, and what was done if the assumptions were not met.	Section 2.11

**Experimental animals**	8	a. Provide species-appropriate details of the animals used, including species, strainand substrain, sex, age or developmental stage, and, if relevant, weight.<br>b. Provide further relevant information on the provenance of animals, health/immune status, genetic modification status, genotype, and any previous procedures.	Section 2.2
**Experimental procedures**	9	For each experimental group, including controls, describe the procedures in enoughdetail to allow others to replicate them, including:<br>a. What was done, how it was done and what was used.<br>b. When and how often.<br>c. Where (including detail of any acclimatization periods).<br>d. Why (provide rationale for procedures).	Section 2.2
**Results**	10	For each experiment conducted, including independent replications, report:<br>a. Summary/descriptive statistics for each experimental group, with a measure ofvariability where applicable (e.g. mean and SD, or median and range).<br>b. If applicable, the effect size with a confidence interval.	Section 3.

The Recommended Set			
These items complement the Essential 10 and add important context to the study. Reporting the items in both sets represents best practice.			
**Item**		**Recommendation**	**Section/line number, or reason for not reporting**
**Abstract**	11	Provide an accurate summary of the research objectives, animal species, strain<br>and sex, key methods, principal findings, and study conclusions.	Lines 37-52
**Background**	12	a. Include sufficient scientific background to understand the rationale and<br>context for the study, and explain the experimental approach.<br>b. Explain how the animal species and model used address the scientific<br>objectives and, where appropriate, the relevance to human biology.	Section 2
**Objectives**	13	Clearly describe the research question, research objectives and, where appropriate, specific hypotheses being tested.	Section 1<br>Lines 75-81
**Ethical statemen t**	14	Provide the name of the ethical review committee or equivalent that has approvedthe use of animals in this study, and any relevant licence or protocol numbers (if applicable). If ethical approval was not sought or granted, provide a justification.	Lines 83-84
**Housing and husbandry**	15	Provide details of housing and husbandry conditions, including any environmentalenrichment.	Section 2.3<br>Lines 114-123
**Animal care andmonitoring**	16	a. Describe any interventions or steps taken in the experimental protocols toreduce pain, suffering and distress.<br>b. Report any expected or unexpected adverse events.<br>c. Describe the humane endpoints established for the study, the signs that weremonitored and the frequency of monitoring. If the study did not have humaneendpoints, state this.	Section 2.<br>Section 3
**Interpretation/ scientific implications**	17	a. Interpret the results, taking into account the study objectives and hypotheses,<br>current theory and other relevant studies in the literature.<br>b. Comment on the study limitations including potential sources of bias, limitations of the animal model, and imprecision associated with the results.	Section 3 and 4
**Generalizability /translation**	18	Comment on whether, and how, the findings of this study are likely to generalize to other species or experimental conditions, including any relevance to human biology (where appropriate).	Not applicable
**Protocol registratio n**	19	Provide a statement indicating whether a protocol (including the research question, key design features, and analysis plan) was prepared before the study,and if and where this protocol was registered.	Not applicable
**Data access**	20	Provide a statement describing if and where study data are available.	Supplementary material 9 – Data sheet
**Declaration of interests**	21	a. Declare any potential conflicts of interest, including financial and non-financial.<br>If none exist, this should be stated.	Included in submission<br>Included in submission

	b. List all funding sources (including grant identifier) and the role of the funder(s)<br>in the design, analysis and reporting of the study.	
