# Supplementary material for: Validation of the Portuguese Version of the Short-Form Glasgow Composite Measure Pain Scale (CMPS-SF) According to COSMIN and GRADE Guidelines
Source: Animals (Basel). 2024 Mar 8;14(6):831. doi: 10.3390/ani14060831 (PMC10967594; doi:10.3390/ani14060831)
Supplement: Supplementary file 1 [file animals-14-00831-s001.zip › Supplementary files/Supplementary material 3 - Certificate.pdf]

# CERTIFICATE

We hereby certify that the translation from English into Protuguese (Brazil) of the document "Glasgow Dog Scale.docx" has been carried out in accordance with **UNE EN 15038:2006** (European standard for provision of translation services).

Mondo Agit LLC

By: Jacobo Guzmán Olalde
Senior Project Manager
Mondo Agit LLC

Name: Jacobo Guzmán
Title: Senior Project Manager
Date: 1 June 2023

Mondo Agit LLC
2880 W OAKLAND PARK BLVD
SUITE 225C
OAKLAND PARK, FL 33311 (USA)
EIN: 86-3141245
