# Supplementary material for: Validation of the Portuguese Version of the Short-Form Glasgow Composite Measure Pain Scale (CMPS-SF) According to COSMIN and GRADE Guidelines
Source: Animals (Basel). 2024 Mar 8;14(6):831. doi: 10.3390/ani14060831 (PMC10967594; doi:10.3390/ani14060831)
Supplement: Supplementary file 1 [file animals-14-00831-s001.zip › Supplementary files/Supplementary material 4 - Portuguese version of CMPS-SF.pdf]

Supplementary 4. Portuguese version of CMPS-SF [10].

FORMULÁRIO RESUMIDO DA ESCALA DE DOR COMPOSTA DE GLASGOW

Nome do cão ______________________________

Número do hospital________**Data** / / **Hora**

Cirurgia Sim/Não (eliminar se necessário)

Procedimento ou doença____

Nas seções abaixo, circular a pontuação apropriada em cada lista e some-as para obter a pontuação total.

A. Observe o cão de fora do canil

Como está o cão?

(i)

Quieto	0
Chorando ou lamuriando	1
Gemendo	2
Gritando	3

(ii)

Ignorando qualquer ferida ou área dolorida	0
Olhando para a ferida ou área dolorida	1
Lambendo a ferida ou a área dolorida	2
Esfregando a ferida ou a área dolorida	3
Mordendo a ferida ou área dolorida	4

No caso de fraturas da coluna vertebral, pélvicas ou de múltiplos membros, ou quando for necessária assistência para auxiliar a locomoção, não avaliar a seção **B** e prosseguir para a seção **C**. *Marque se este for o caso* ☐ e depois siga para a **C**.

B. Coloque a guia no cão e retire-o do canil.

Quando o cão se levanta/caminha, ele parece...

(iii)

Normal	0
Manco	1
Lento ou relutante	2
Rijo	3
Relutante em mover-se	4

C. Se tiver uma ferida ou área dolorida, incluindo o abdômen, aplique uma pressão suave ao redor (5 cm) do local.

Ao ser tocado, o cão...

(iv)

Não tem reação	0
Olha ao redor	1
Esquiva	2
Rosna ou protege a ferida	3
Morde	4
Chora	5

D. Geral

Como está o cão?

(v)

Feliz e contente ou feliz e animado	0
Quieto	1
Indiferente ou impassível ao ambiente	2
Nervoso, ansioso ou com medo	3
Deprimido ou indiferente à estimulação	4

Como está o cão?

(vi)

Confortável	0
Ansioso/inquieto	1
Agitado	2
Encolhido ou tenso	3
Rijo	4

© Universidade de Glasgow

Pontuação total (i+ii+iii+iv+v+vi) = __
