# Supplementary material for: Validation of the Portuguese Version of the Short-Form Glasgow Composite Measure Pain Scale (CMPS-SF) According to COSMIN and GRADE Guidelines
Source: Animals (Basel). 2024 Mar 8;14(6):831. doi: 10.3390/ani14060831 (PMC10967594; doi:10.3390/ani14060831)
Supplement: Supplementary file 1 [file animals-14-00831-s001.zip › Supplementary files/Supplementary material 5 - Demographic data.pdf]

Supplementary 5. Demographic description of the dogs in the control (n = 10), orthopedic (n = 20) and soft tissue (n = 22) groups.

Animal	Group	Breed	Name	Surgical intervention
1	OG	Pug	Udy	Colocephalectomy R
2	OG	Cross breed	Scooby	Osteosynthesis R/U R
3	OG	Lhasa apso	Batatinha	Colocephalectomy R
4	OG	Cross breed	Jack	Patelopexy L
5	OG	Lhasa	Pidi	Patelopexy R
6	OG	Cross breed	Titio	Tibial Osteosynthesis R
7	OG	Cross breed	Diogo	Umeral osteosynthesis R
8	OG	Cross breed	Pingo	Tibial Osteosynthesis L
9	OG	Cross breed	Frida	Elbow arthrodesis L
10	OG	Cross breed	Pelé	Carpal arthrodesis R
11	OG	Pitbull	Meg	Colocephalectomy R
12	OG	Cross breed	Toddy	Ileo-femoral suture R
13	OG	Pequinês	Topêra	Trochleoplasty R
14	OG	Cross breed	Meg	Femoral osteosynthesis R
15	OG	Cross breed	Maisena	Trochleoplasty L
16	OG	Cross breed	Nina	Tibial Osteosynthesis R
17	OG	Cross breed	Madruguinha	Colocephalectomy L
18	OG	Pitbull	Duque	Metatarsal-phalangeal arthrodesis R
19	OG	Pinscher	Chiquinho	Colocephalectomy L
20	OG	Poodle	Meg	Osteosynthesis de R/U R
21	STG	Pinscher	Mimosa	Mastectomy and OVH
22	STG	Pitbull	Pedrita	OVH
23	STG	Cross breed	Sky	OVH
24	STG	Cross breed	Lilica	Mastectomy and OVH
25	STG	Pitbull	Athena	OVH
26	STG	Lhasa apso	Nick	Cystotomy
27	STG	Doberman	Dara	Mastectomy and OVH
28	STG	Poodle	Marie	Mastectomy and OVH
29	STG	Border collie	Jade	OVH
30	STG	York	Cindy	Cystotomy
31	STG	Cross breed	Jack	Orchiectomy
32	STG	Rottweiler	Alexia	Mastectomy
33	STG	Cross breed	Amarelo	Orchiectomy
34	STG	Cross breed	Poka Sombra	Exploratory laparotomy
35	STG	Pitbull	Balada	OVH
36	STG	Teckel	Neneca	Mastectomy and OVH
37	STG	Pitbull	Tranquera	Mastectomy and OVH
38	STG	Poodle	Luna	Mastectomy
39	STG	Cross breed	Marrom	Orchiectomy
40	STG	Poodle	Pitty	Mastectomy
41	STG	Cross breed	Lily	Flap and dermal graft

42	STG	Cross breed	Tuti	Mastectomy and OVH
43	CG	Cross breed	Alvorada	Controll
44	CG	Cross breed	Remela	Controll
45	CG	Border collie	Ayka	Controll
46	CG	Pitbull	Chayra	Controll
47	CG	Lhasa apso	Alvin	Controll
48	CG	Akita	Athena	Controll
49	CG	Cocker	Coisinha	Controll
50	CG	Cross breed	Lara	Controll
51	CG	York	Mel	Controll
52	CG	Cross breed	Milka	Controll

Interpretation: CG is Control group, OG is orthopedic group, STG is soft tissue group, R is right side and L is left side and OVH is ovariosalpingohisterectomy.
