# Supplementary material for: Validation of the Portuguese Version of the Short-Form Glasgow Composite Measure Pain Scale (CMPS-SF) According to COSMIN and GRADE Guidelines
Source: Animals (Basel). 2024 Mar 8;14(6):831. doi: 10.3390/ani14060831 (PMC10967594; doi:10.3390/ani14060831)
Supplement: Supplementary file 1 [file animals-14-00831-s001.zip › Supplementary files/Supplementary material 7 - Table S1 Inter-rater reliability.pdf]

Table S1. Interobserver reliability of four evaluators for indication of RA, unidimensional scales (VAS, NRS, and SDS), and composite pain scales in dogs (CMPS-SF), obtained by analyzing 208 videos from four perioperative moments (M1, M2, M3, and M4) of dogs undergoing orthopedic (n = 20) or soft tissue surgeries (n = 22) and negative control group (n = 10), between two phases, 21 days apart.

Statistical test	Variables	PhD student x Ethologist	PhD student x MSc student	PhD student x Student	Ethologist x MSc student	Ethologist x Student	MSc student x Student
**Kw (CI95%)**	Analgesic rescue	**0.6 (0.52-0.68)**	**0.53 (0.45-0.61)**	**0.51 (0.42-0.6)**	0.42 (0.34-0.51)	**0.54 (0.46-0.62)**	0.4 (0.31-0.5)
	Numerical rating scale	0.45 (0.38-0.52)	**0.6 (0.54-0.66)**	0.41 (0.34-0.49)	0.29 (0.22-0.36)	**0.68 (0.52-0.67)**	0.29 (0.22-0.36)
	Simples descriptive scale	**0.52 (0.43-0.6)**	**0.54 (0.47-0.61)**	**0.53 (0.45-0.61)**	0.33 (0.24-0.41)	**0.6 (0.52-0.67)**	0.39 (0.31-0.46)
**ICC (CI95%)**	Visual analogue scale	**0.71 (0.65-0.76)**	**0.77 (0.72-0.81)**	**0.7 (0.58-0.72)**	0.36 (0.3-0.45)	**0.75 (0.69-0.79)**	**0.64 (0.56-0.7)**
**Kw (CI95%)**	A(i) – Spontaneous behavior	**0.58 (0.46-0.69)**	**0.53 (0.41-0.63)**	**0.52 (0.4-0.63)**	**0.52 (0.43-0.59)**	**0.56 (0.43-0.68)**	**0.56 (0.47-0.64)**
	A(ii) – Attention to wound	**0.59 (0.4-0.74)**	0.37 (0.21-0.55)	0.32 (0.11-0.53)	0.46 (0.28-0.6)	0.56 (0.27-0.77)	0.3 (0.13-0.47)
	B (iii) – Mobility	**0.6 (0.5-0.68)**	**0.62 (0.51-0.71)**	0.46 (0.34-0.57)	**0.52 (0.4-0.63)**	**0.71 (0.58-0.81)**	0.46 (0.33-0.59)
	C (iv) – Response to touch	**0.65 (0.55-0.73)**	**0.77 (0.69-0.83)**	**0.76 (0.69-0.84)**	**0.72 (0.63-0.79)**	**0.74 (0.66-0.82)**	**0.83 (0.76-0.88)**
	D (v) – Demeanor	0.28 (0.2-0.36)	0,48 (0.39-0.56)	**0.54 (0.47-0.6)**	0.34 (0.26-0.43)	0.27 (0.19-0.35)	**0.52 (0.43-0.6)**
	D (vi) – Posture	0.41 (0.32-0.49)	0.37 (0.29-0.46)	0.29 (0.19-0.4)	0.31 (0.21-0.4)	0.39 (0.28-0.49)	0.33 (0.22-0.43)
**ICC (CI95%)**	**Total CMPS-SF**	**0.8 (0.76-0.83)**	**0.83 (0.8-0.86)**	**0.8 (0.77-0.85)**	**0.78 (0.73-0.81)**	**0.82 (0.78-0.85)**	**0.83 (0.79-0.86)**

Interpretation: 0.81-1.0 very good; 0.61-0.80 good; 0.41-0.60 moderate; 0.21-0.40 reasonable; <0.20 poor [35]. CMPS-SF is Short-Form Composite Pain Score [10], Kw is Weighted Kappa Coefficient, ICC is Intraclass Correlation Coefficient, [CI95% (Min-Max): 95% confidence interval (minimum-maximum values)]. In bold are the values considered acceptable by the adapted GRADE criteria [14,15].
